# *ABCG2* in peptic ulcer: gene expression and mutation analysis

**DOI:** 10.1007/s13353-015-0327-0

**Published:** 2015-11-17

**Authors:** Aleksandra Salagacka-Kubiak, Marta Żebrowska, Agnieszka Wosiak, Mariusz Balcerczak, Marek Mirowski, Ewa Balcerczak

**Affiliations:** 1 Laboratory of Molecular Diagnostics and Pharmacogenomics, Department of Pharmaceutical Biochemistry and Molecular Diagnostics, Interfaculty Cathedral of Laboratory and Molecular Diagnostics, Medical University of Lodz, Muszynskiego 1, 90-151 Lodz, Poland; 2 Department of Surgery, District Hospital, Zachodnia 6, 99-100 Leczyca, Poland

**Keywords:** *ABCG2*, C421A, Expression, *Helicobacter pylori*, Peptic ulcer, Polymorphism

## Abstract

The aim of this study was to evaluate the participation of polymorphism at position C421A and mRNA expression of the *ABCG2* gene in the development of peptic ulcers, which is a very common and severe disease. ABCG2, encoded by the *ABCG2* gene, has been found inter alia in the gastrointestinal tract, where it plays a protective role eliminating xenobiotics from cells into the extracellular environment. The materials for the study were biopsies of gastric mucosa taken during a routine endoscopy. For genotyping by polymerase chain reaction-restriction fragment length polymorphism (PCR-RFLP) at position C421A, DNA was isolated from 201 samples, while for the mRNA expression level by real-time PCR, RNA was isolated from 60 patients. The control group of healthy individuals consisted of 97 blood donors. The dominant genotype in the group of peptic ulcer patients and healthy individuals was homozygous CC. No statistically significant differences between healthy individuals and the whole group of peptic ulcer patients and, likewise, between the subgroups of peptic ulcer patients (infected and uninfected with *Helicobacter pylori*) were found. *ABCG2* expression relative to *GAPDH* expression was found in 38 of the 60 gastric mucosa samples. The expression level of the gene varies greatly among cases. The statistically significant differences between the intensity (*p* = 0.0375) of *H. pylori* infection and *ABCG2* gene expression have been shown. It was observed that the more intense the infection, the higher the level of *ABCG2* expression.

## Introduction

Peptic ulcer (PU) is a disease with a high percentage of morbidity. Due to its complications such as bleeding, pyloric stenosis and life-threatening perforations occurring in 10–20 % of patients, peptic ulcer diseases are a serious condition. According to the Demographic Yearbook of Poland, in 2012, 1886 people died because of duodenal and gastric ulcer in Poland (Dmochowska 2014; Malfertheiner et al. 2009; Ramakrishnan and Salinas 2007; Szyca et al. 2012; Thorsen et al. 2013).

The development of peptic ulcer may be influenced not only by environmental factors, e.g. cigarette smoking, alcohol consumption, diet high in salt, steroids and non-steroidal anti-inflammatory drugs, *Helicobacter pylori* infection and stress, but also a patient’s genetic predisposition (Schabowski 2002; Thorsen et al. 2013). It concerns changes in the genes physiologically involved in the protection of gastric mucosa.

One of the plausible genetic factors contributing to the development of peptic ulcer disease could be changes in the *ABCG2* gene. It encodes protein named ABCG2 with 655 amino acids (previously BCRP, breast cancer resistance protein), which is a half-transporter belonging to the ABC transporters superfamily. As for all proteins in this family, ABCG2 uses energy from ATP hydrolysis to transport substrates. The protein has been found inter alia in the brain, blood–brain barrier, prostate, ovaries, testes, placenta, adrenal gland and gastrointestinal tract, where it plays a protective role eliminating xenobiotics from cells into the extracellular environment. In the digestive tract, *ABCG2* is localised in the apical membrane of cells, which confirms the hypothesis of its protective role by limiting accumulation in cells/organs of harmful xenobiotics. On the other hand, one loss of its function could promote peptic ulcer development (Mao and Unadkat 2005; Mo and Zhang 2012; Liu and Liu 2013).

The *ABCG2* gene is highly polymorphic. So far, over 40 single nucleotide polymorphisms (SNPs) have been found (Iida et al. 2002). SNPs may affect the level of mRNA expression, which leads to a change/loss of ABCG2 function. This could result in the accumulation of xenobiotics in cells and, thus, in the increased risk of peptic ulcer disease development. One of such polymorphisms is the SNP substitution of C to T at position 421. It is a non-synonymous polymorphism leading to a change of glutamine into lysine at position 141 in protein (Q141K) (Cusatis et al. 2006). The *ABCG2* gene polymorphism at position 421 is associated with a lower expression and a lower transport activity of encoded protein (Imai et al. 2002; Kondo et al. 2004; Mizuarai et al. 2004).

Fehér et al. (2013) found a significantly increased susceptibility to Alzheimer’s disease [odds ratio (OR) = 1.741, 95 % confidence interval (CI): 1.075–2.819, *p* = 0.024) associated with the *ABCG2* C/C genotype when compared with the variant allele containing genotypes CA and AA as the control group. In pancreatic cancer, such a correlation was not found (Pang et al. 2014).

The aim of this study was to evaluate the participation of polymorphism at position C421A and the mRNA expression level of the *ABCG2* gene in the development of peptic ulcer. To the best of our knowledge, this area has not been explored so far.

## Materials and methods

The materials for the study were biopsies of gastric mucosa taken during endoscopy from patients with peptic ulcer diagnosed at the Department of Surgery, District Hospital, Leczyca, Poland. The obtained samples were residual material remaining after a routine diagnostics.

A small amount of biopsies material did not allow the simultaneous isolation of two nucleic acids (DNA and RNA) from one sample. Therefore, in order to analyse both polymorphism at position C421A and mRNA expression, the group of patients with peptic ulcer disease diagnosed at the same time in the Department of Surgery, District Hospital, Leczyca, Poland was divided into two investigated groups. The first group consisted of 201 patients (129 females; 72 males; median age of the group: 53 years), from whom DNA was isolated in order to genotype C421A of *ABCG2* (investigated group I). The other group consisted of 60 patients (34 females; 26 males; median age of the group: 60 years), from whom RNA was isolated in order to assess the mRNA expression level (investigated group II). Patients who were treated with non-steroidal anti-inflammatory drugs were excluded from the study.

The group of healthy individuals consisted of 97 blood donors (58 females; 39 males; median age of the group: 33 years) from the local blood bank, and they geographically and ethnically matched the group of patients with peptic ulcer.

Data concerning exposure to carcinogens in patients and healthy individuals were not available. The investigation was in accordance with the principles of the Declaration of Helsinki, and was approved by the Ethical Committee of the Medical University of Lodz (RNN/285/13/KE; RNN/195/13/KE). All individuals included in the study gave informed consent.

### DNA and RNA isolation

DNA and RNA from biopsies of gastric mucosa collected during a routine endoscopy were isolated according to the Genomic DNA Mini and Total RNA Mini protocols, respectively (A&A Biotechnology, Poland). The purity and concentration of DNA and RNA samples were assessed spectrophotometrically. Until analysis, DNA and RNA samples had been stored at −20 °C and −76 °C, respectively.

### Genotyping of C421A

#### PCR

For the studied polymorphism, polymerase chain reaction (PCR) was performed in accordance with the AccuTaq™ LA DNA Polymerase Kit protocol (Sigma Aldrich, Germany). The mixture for PCR consisted of 10× *AccuTaq* buffer, 1.5 mM of MgCl_2_, 0.5 μM of each primer (C421A F 5′-ATGTTGTGATGGGCACTCTG-3′; C421A R 5′-TGCTGATCATGATGCTTTCAG-3′), 0.2 mM of each dNTP, 0.5U of *AccuTaq* LA DNA Polymerase, 50 ng of DNA template and distilled water to a final volume of 20 μL. In every experiment, a negative control was included. Products of the PCR reactions were assessed by electrophoresis in 2 % agarose gel. Reaction products for the investigated SNP had a size of 184 bp.

#### RFLP

Genotyping of C421A was performed by restriction fragment length polymorphism (RFLP). Amplified DNA fragments for the SNP on position C421A were digested by MseI (New England Biolabs, USA) for 16 h at 30 °C. Genotypes were identified by electrophoresis of amplified DNA fragments after digestion by restriction enzyme (two bands of 84 and 100 bp for genotype CC; three bands of 36, 64 and 84 bp for genotype AA; four bands of 36, 64, 84 and 100 bp for genotype CA).

### Expression of *ABCG2* mRNA

#### Reverse transcription

A total cellular RNA was transcribed into complementary DNA (cDNA) in accordance with the High Capacity cDNA Reverse Transcription Kit (Applied Biosystems, USA). The final concentration of RNA in the reaction mixture was 0.01 μg/μL. Synthesised cDNA was stored at −20 °C until analysis. As a reference gene, *GAPDH* was used. Only the samples which showed the presence of PCR product for the *GAPDH* gene were included in the analysis.

#### PCR

For qualitative analysis of the mRNA expression of the reference gene *GAPDH*, a PCR assay was performed in accordance with the *AccuTaq*™ LA DNA Polymerase Kit protocol (Sigma Aldrich, Germany). The mixture for PCR consisted of 10× *AccuTaq* buffer, 1 mM of MgCl_2_, 0.5 μM of each primer (*GAPDH* gene: F 5′-TGGTATCGTGGAAGGACTCATGAC-3′, R 5′-ATGCCAGTGAGCTTCCCGTTCAGC-3′), 0.2 mM of each dNTP, 0.5U of *AccuTaq* LA DNA Polymerase, 0.2 μg of cDNA template and distilled water to a final volume of 20 μL. In every experiment, a negative control was included. Products of the PCR reactions were assessed by electrophoresis in 2 % agarose gel. The reaction product for *GAPDH* had a size of 257 bp.

#### Real-time PCR

Quantification assessment of *ABCG2* (investigated gene) and *GAPDH* (reference gene) mRNA was performed by real-time PCR using a Rotor-Gene™ 6000 (Corbett Research, Germany), according to the KAPA SYBR® FAST qPCR Kit Master Mix (2X) Universal protocol (Kapa Biosystems, USA). The reaction mixture for both genes consisted of 10 μL KAPA SYBR FAST qPCR Master Mix (2X), 0.4 μL of each primer (*ABCG2* gene: F 5′-ATGTCAACTCCTCCTTCTAC-3′; R 5′-AATGATCTGAGCTATAGAGGC-3′; *GAPDH* gene: F 5′-TGGTATCGTGGAAGGACTCATGAC-3′, R 5′-ATGCCAGTGAGCTTCCCGTTCAGC-3′), 1.5 μL of cDNA and distilled water up to a final volume of 20 μL. The reactions for *ABCG2* and *GAPDH* were carried out in separate tubes. Samples were tested in triplicate, and the mean of the obtained Ct values for both *ABCG2* and *GAPDH* was calculated. In each experiment, a negative control, also tested in triplicate, was included. For estimation of the kinetic PCR reaction efficiency, the standard curves for both genes, by four serial 10-fold dilutions of a quantified PCR product (obtained with the same primers as those used in the quantification mRNA analysis), were constructed. The efficiencies were calculated from the slopes of the standard curves according to the equation E = 10^[−1/slope]^ − 1. Because the efficiencies for both genes were different (*ABCG2* 122 %; *GAPDH* 107 %), the Pfaffl method was used to calculate relative changes in gene expression (Livak and Schmittgen 2001; Tyburski et al. 2008). The mean Ct values for the *ABCG2* and *GAPDH* genes for all investigated samples were utilised as calibrators.

### Statistical analysis

All statistical analyses were performed using STATISTICA 10 (StatSoft, Inc., 2011). The χ^2^ test, χ^2^ test with Yates’ correction and V^2^ test were applied to evaluate conformity between the observed and expected genotype frequencies according to the Hardy–Weinberg rule and to determine the significance of differences in allele and genotype frequencies between patients and controls and between subgroups of patients. The collected quantitative data were tested to check for conformity with a normal distribution on the basis of the Shapiro–Wilk test. Because of the lack of normality of *ABCG2* expression values, decimal logarithm of the expression level was used for statistical analysis. Student’s *t*-test and analysis of variance (ANOVA) were applied to assess the differences in the mean values of the *ABCG2* expression between the subgroups of patients. In all conducted tests, a *p*-value < 0.05 was assumed to be significant.

## Results

### Genotyping of *ABCG2*

All 201 biopsy specimen of gastric mucosa (peptic ulcer patients; investigated group I) and 97 blood samples (healthy individuals) for the SNP at position C421A of the *ABCG2* gene were successfully analysed. All genotypes for the investigated polymorphism in both peptic ulcer patients and healthy individuals were in Hardy–Weinberg equilibrium.

Firstly, genotype and allele frequencies for the polymorphism at position C421A between peptic ulcer patients (investigated group I) and healthy individuals were compared (Table 1). For both genotype and allele of the studied SNP, no statistically significant differences between peptic ulcer patients and healthy individuals were found (*p* = 0.7845 and 0.9370, respectively). The dominant genotype in both groups was homozygous CC (97.5 % for peptic ulcer and 97.9 % for healthy individuals), whereas genotype AA occurred only in the group of patients with peptic ulcer (0.5 %).

**Table 1:** Comparison of C421A *ABCG2* allele and genotype frequencies between peptic ulcer (investigated group I) and healthy individuals

	Peptic ulcer cases, *n* = 201	Healthy individuals, *n* = 97	*p*-Value
CC	196 (97.5 %)	95 (97.9 %)	0.7845^a^
CA	4 (2.0 %)	2 (2.1 %)	
AA	1 (0.5 %)	0 (0.0 %)	
C	396 (98.5 %)	192 (99.1 %)	0.9370^b^
A	6 (1.5 %)	2 (1.0 %)	
HWE: *p*	0.4960^a^	0.6134^b^	

^a^χ^2^ test

^b^χ^2^ test with Yates’ correction

Secondly, on the basis of the results of rapid urease tests, the group of peptic ulcer patients were divided into two subgroups: patients infected with *H. pylori* and patients uninfected with this bacterium. The frequency of genotypes and alleles of the SNP C421A for these two subgroups was compared. No statistically significant differences for the studied polymorphism were observed (*p* = 0.3591 and 0.2127, respectively). Nevertheless, it is noteworthy that allele A occurred more frequently in the subgroup of patients uninfected with *H. pylori* than in the infected subgroup (2.5 % and 0.5 %, respectively). The results are shown in Table 2.

**Table 2:** Comparison of C421A *ABCG2* allele and genotype frequencies between the subgroup of patients infected with *Helicobacter pylori* and the subgroup of patients uninfected with *H. pylori*

	All peptic ulcer cases		*p*-Value
	Infected, *n* = 101	Uninfected, *n* = 100	
CC	100 (99.0 %)	96 (96.0 %)	0.3591^a^
CA or AA	1 (1.0 %)	4 (4.0 %)	
C	201 (99.5 %)	195 (97.5 %)	0.2127^a^
A	1 (0.5 %)	5 (2.5 %)	

^a^χ^2^ test with Yates’ correction

In the last step of the analysis, the subgroups of patients infected and uninfected with *H. pylori* were divided further according to gender. First, a comparison of the incidence of genotype and allele of polymorphism C421A between the subgroup of infected women and a subgroup of women not infected with *H. pylori* was conducted. No statistically significant differences were found (*p* = 0.3524 and 0.2127, respectively). However, allele A occurred more frequently in the subgroup of uninfected women than in the subgroup of women infected with *H. pylori*. This observation was similar to the results obtained in the whole subgroup of patients uninfected with this bacterium. On the other hand, among men, no genotype AA was found. Therefore, no analogous analysis was possible.

### Expression of mRNA *ABCG2*

Initially, a qualitative analysis of the *GAPDH* gene was performed to check the reverse transcription of the procedure. In all tested samples, the expression of *GAPDH* was present. *ABCG2* expression was found in 38 of the 60 gastric mucosa samples. There were no statistical differences between gender, age and presence of the investigated gene expression (Table 3). There was also no correlation between the presence of *H. pylori* infection and the presence of gene expression, but in a more intensive infection, *ABCG2* expression appeared more frequently (*p* = 0.0324, Table 3).

**Table 3:** Presence of *ABCG2* expression according to gender, age, presence and intensity of *H. pylori* infection

Feature	*ABCG2* expression		*p*-Value
	Absent	Present	
Gender			0.7749^a^
Female	13 (59.1 %)	21 (55.3 %)	
Male	9 (40.9 %)	17 (44.7 %)	
Age			0.7749^a^
<60 years	9 (40.9 %)	17 (44.7 %)	
>60 years	13 (59.1 %)	21 (55.3 %)	
*H. pylori* infection			0.1608^a^
Absent	11 (50.0 %)	26 (68.4 %)	
Present	11 (50.0 %)	12 (31.6 %)	
*H. pylori* infection intensity			
Low	9 (81.8 %)	5 (41.7 %)	**0.0324** ^b^
Medium	0 (0.0 %)	4 (33.3 %)	
High	2 (18.2 %)	3 (25.0 %)	

^a^V^2^ test

^b^χ^2^ test

**Fig. 1:**
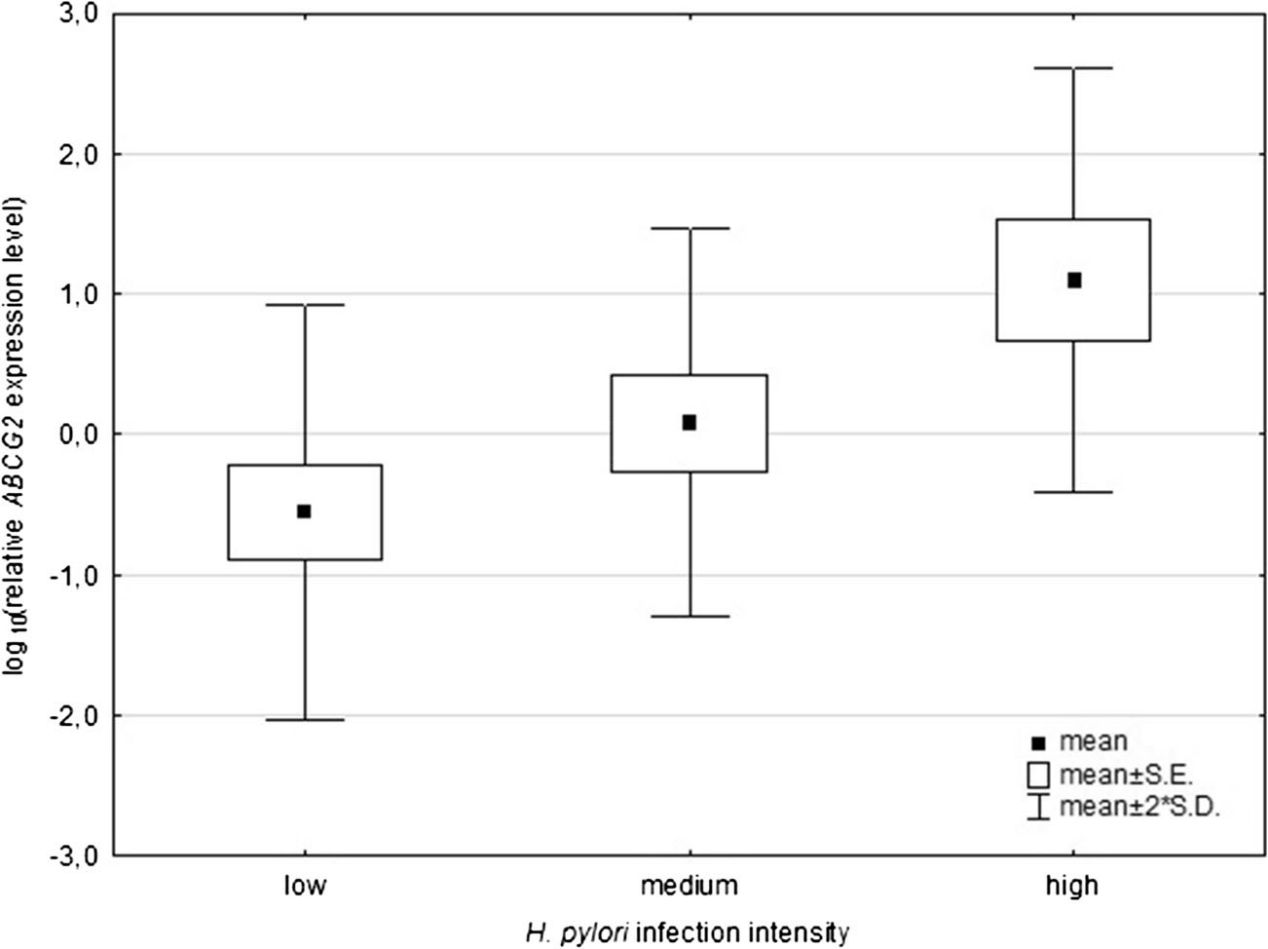
Relative expression level of *ABCG2* according to the *Helicobacter pylori* infection intensity

The expression level of *ABCG2* relative to *GAPDH* varies greatly among cases. It ranged from −1.64 to 1.75, with a mean value of −0.13 (standard deviation 0.77). There was no association between *ABCG2* expression level and the patient’s gender (*p* = 0.4957). Also, no connection between the level and patient’s age was stated (*p* = 0.5329, Table 4). No difference in the expression level was also stated when it was compared between patients <60 and >60 years old. There was no significant connection between the presence of *H. pylori* infection and the expression level of *ABCG2* (*p* = 0.2864, Table 4). In the *H. pylori*-infected patients divided into three subgroups according the infection intensity, the expression level of the investigated gene differed significantly between the subgroups (*p* = 0.0375, Table 4). The more intense the infection, the higher the level of *ABCG2* expression was observed. The post-hoc analysis showed that there was a significant difference in the expression level between the subgroups of low and high intensity of infection (*p* = 0.0306, Fig. 1).

**Table 4:** Relative *ABCG2* expression level according to gender, age, presence and intensity of *H. pylori* infection

Feature	*n*	Relative *ABCG2* expression level				*p*-Value
		Mean	Min.	Max.	SD	
Gender						0.4957^a^
Female	21	−0.05	−1.28	1.75	0.82	
Male	17	−0.22	−1.64	1.28	0.72	
Age						
<60 years	17	−0.22	−1.28	0.75	0.63	0.5329^a^
>60 years	21	−0.06	−1.64	1.75	0.88	
*H. pylori* infection						
Absent	26	−0.22	−1.64	1.03	0.68	0.2864^a^
Present	12	0.07	−1.19	1.75	0.95	
*H. pylori* infection intensity						
Low	5	−0.55	−1.19	0.67	0.74	**0.0375** ^b^ 0.4272^c^ 0.2158^d^ **0.0306** ^e^
Medium	3	0.08	−0.86	0.75	0.69	
High	4	1.10	0.27	1.75	0.76	

^a^Student’s *t*-test

^b^ANOVA

Tukey’s HSD test: ^c^low vs. medium, ^d^medium vs. high, ^e^low vs. high

## Discussion

ABCG2 was described for the first time in tumour cells, where it produced a significant resistance to chemotherapeutic agents (Doyle et al. 1998). The transporter on cells weakens access of drugs to the cellular target and, thereby, contributes to a reduction in the efficacy of the applied therapy. As ABCG2 has a low substrate specificity, it might contribute to the resistance to structurally diverse anti-cancer drugs, a phenomenon called multidrug resistance (MDR) (Gottesman et al. 2002). However, ABCG2 is present not only in tumour cells, and its function in the development of MDR is a manifestation of the physiological role of this protein.

Protein product of the *ABCG2* gene is located in many normal tissues, including the gastrointestinal tract, where it plays a protective role by removing endogenous and exogenous xenobiotics, i.e. bacterial toxins from cells to the extracellular environment. Any changes in the *ABCG2* gene, such as SNPs or in gene mRNA expression, may influence the protein level and/or its function. That may lead to a loss of its protective function and, thus, increase the risk of peptic ulcer development. Imai et al. showed that the occurrence of allele A of the SNP at position C421A may be associated with a lower expression of the protein in comparison to wild-type C of this polymorphism. It was suggested that the substitution of glutamine by lysine on position 141, as a consequence of the studied SNP, may lead to the increased susceptibility to degradation of the protein. This is a consequence of the fact that lysine and glutamine have different electronic charges, which might alter the tertiary structure of protein (Imai et al. 2002).

So far, to the best of our knowledge, participation of the *ABCG2* gene polymorphism at position C421A and mRNA expression in the peptic ulcer development has not been investigated. All genotypes in the peptic ulcer patients and healthy individuals were in accordance with the Hardy–Weinberg rule, which proves their representativeness of the studied population (Table 1). In this research, the dominant genotype was homozygous CC, which is similar to the results in European Caucasian (de Jong et al. 2004), non-Hispanic Caucasian (Gardner et al. 2008), Hungarian (Fehér et al. 2013) and Sub-African populations (de Jong et al. 2004), but which was different in Han Chinese (Hu et al. 2007), Korean (Kim et al. 2010) and Japan (Imai et al. 2002) populations. Contrary to our results, the incidence of genotype CC in those studies was lower but homozygous AA occurred with a greater frequency than in Caucasian and African populations.

Through the comparison of genotyping results between peptic ulcer patients and healthy individuals, the impact of the investigated polymorphism on peptic ulcer development was estimated. No statistically significant differences for this polymorphism were found (Table 1). Therefore, it can be assumed that the studied polymorphism is not associated with an increased risk of developing peptic ulcer disease. However, the results may confirm a protective role of ABCG2, which may be associated with the presence of the CC genotype of the SNP on position C421A. This genotype is connected with a higher expression and transport activity of protein (Morisaki et al. 2005), which, in turn, will lead to a greater elimination of toxins/xenobiotics from gastric mucosa cells. Sparreboom et al. (2005), in a study on the cell line HEK293, showed that allele C of C421A was responsible for the overexpression of *ABCG2* and decreased cell accumulation of topotecan. On the other hand, HEK293 cells with the allele A variant of the studied SNP showed an increase in the intracellular accumulation of topotecan.

Our hypothesis may be confirmed by the results of the research presented by Gardner at el. (2008), who also demonstrated no statistical significance between polymorphism at position C421A and risk of prostate cancer development, but a higher incidence of the CC genotype was associated with increased survival in patients with this cancer. Zhou et al. (2014) showed that the occurrence of genotype AA and allele A was associated with higher risk of gout development in the male Han Chinese population.

This is the first report concerning *ABCG2* expression in the gastric mucosa of peptic ulcer patients. We found expression of the gene in 63.3 % of analysed samples. Previously, ABCG2 protein was investigated by immunohistochemistry in selected human tissues. Prominent ABCG2 immunostaining was stated in the gastrointestinal tract, with strong apical staining of the epithelium of the small intestine and colon, but no staining in the stomach epithelium was found. *ABCG2*/*PBGD* mRNA expression ratios in this tissue measured by qualitative RT-PCR was relatively low (Maliepaard et al. 2001). In the presented research, in samples positive for *ABCG2* expression, the level of expression differed highly among cases. As ABCG2 protein was not stated by others (Maliepaard et al. 2001) in the epithelium of the stomach, the observed expression of *ABCG2* could derive from the capillaries endothelial cells, where expression of the protein was shown (Diestra et al. 2002; Maliepaard et al. 2001).

ABCG2 expression is regulated by hormones. The *ABCG2* promoter estrogen and progesterone response elements were described. It has been shown (Ee et al. 2004; Mao 2008) that these hormones increase the level of mRNA or BCRP, which could suggest that some gender-dependent differences in the *ABCG2* expression level exist. However, we found no association between gender and the presence of *ABCG2* expression. Similarly, no connection between gender and level of the *ABCG2* expression was stated. It remains in agreement with research results obtained by Gutmann et al. (2005), who showed that the expression of *ABCG2* mRNA was not significantly different between males and females, in neither the duodenum and the terminal ileum nor in the different colonic segments of the human gastrointestinal tract.

Recently, we have shown that the expression level of another gene belonging to the ABC transporter superfamily, namely *ABCB1*, is associated with age in peptic ulcer patients (Jażdżyk et al. 2014). *ABCB1* expression was higher in older patients. There is also some evidence that *ABCG2* could exhibit a differential level of the expression depending on age. Kawase et al. (2015) stated that the mRNA level of *ABCG2* decreased with age in the liver of female rats (but not in males). On the other hand, when the expression of *ABCG2* by liquid chromatography coupled with tandem mass spectrometry in the human liver was investigated, the expression was not associated with age, gender or mRNA expression (Prasad et al. 2013). In the presented study, neither the presence nor the level of the expression of *ABCG2* were connected with age and gender. Considering this and the previously published contradictory findings, the existence of a link between *ABCG2* expression and age and gender remains unanswered.

Petrovic et al. (2015) examined the effect of chorioamnionitis, a bacterial intra-amniotic infection, on the expression of the *ABCG2* gene and related protein in human placenta. Expression of both the gene and the protein was downregulated in placentas from women with infection relative to healthy controls. In our study, the impact of *H. pylori* infection on mRNA expression of the transporter in gastric mucosa was analysed. Neither the presence nor the level of expression of *ABCG2* were connected with the presence of *H. pylori* infection in the investigated peptic ulcer patients. However, when the subgroup of infected patients was analysed, the presence of expression was stated to be significantly more frequent in patients of medium or high intensity of *H. pylori* infection in relation to those of low intensity of infection. Moreover, it was discovered that the more intense the infection, the higher the mean value of the expression. The obtained results could suggest that *H. pylori* could potentiate the expression of the investigated gene. In the above-mentioned research, Petrovic et al. (2015) showed that, in the placenta of infected woman, the *ABCB1* expression level correlates with the expression level of interleukin-6 (IL-6), interleukin-1β (IL-1β) and tumour necrosis factor-α (TNF-α), which could indicate the involvement of these cytokines in expression regulation of the gene. Evseenko et al. (2007) showed that the treatment of primary term trophoblasts with TNF-α and IL-1β significantly decreased *ABCG2* protein and mRNA expression, but IL-6 had no significant effect. On the other hand, epidermal growth factor (EGF) and insulin-like growth factor II significantly increased *ABCG2* protein and mRNA expression. It could also be speculated that *ABCG2* expression in the mucosa of peptic ulcer patients could be under an influence of inflammatory cytokine. However, it should be confirmed using a histopathological assessment of *H. pylori* density and inflammation degree in a larger group of patients.

The aim of this study was to evaluate the participation of polymorphism at position C421A and mRNA expression of the *ABCG2* gene in the development of peptic ulcer.

## Conclusion

Based on these studies, it can be concluded that the *ABCG2* gene has no connection with the development of peptic ulcers, although *Helicobacter pylori* infection may lead to increased expression levels of *ABCG2* mRNA, because, with the more intense infection with bacterium, a higher level of *ABCG2* expression was observed.

On the other hand, in this study, because of the small amounts of biopsies material studied, patients with peptic ulcer were divided into two investigated groups and the analyses of C421A single nucleotide polymorphism (SNP) and mRNA expression of *ABCG2* were performed independently of each other. However, as the literature suggests, *ABCG2* expression is largely determined by genetic polymorphisms; thus, it would be beneficial to compare, in the future, the results of genotyping and expression data in the same group of patients with peptic ulcer.
